# Reliability of the 2024 AMA Guides’ Enhanced Methodology for Rating Spine and Pelvis Impairment

**DOI:** 10.3390/jcm14082702

**Published:** 2025-04-15

**Authors:** J. Mark Melhorn, Barry Gelinas, Douglas W. Martin, Kurt T. Hegmann, Matthew S. Thiese

**Affiliations:** 1 Department of Orthopaedics, University of Kansas School of Medicine Wichita, Wichita, KS 67214, USA; 2 International Academy of Independent Medical Evaluators, Vancouver, WA 98683, USA; barry@bgelinasmddc.com; 3 CNOS Occupational Medicine, Dakota Dunes, SD 57049, USA; douglaswaynemartin7@gmail.com; 4 Rocky Mountain Center for Occupational and Environmental Health, University of Utah, Weber State University, Ogden, UT 84403, USA; kurt.hegmann@hsc.utah.edu (K.T.H.); matt.thiese@hsc.utah.edu (M.S.T.)

**Keywords:** AMA, spine, impairment, rating, accuracy, reliability, use

## Abstract

**Background/Objectives**: This study aims to assess the ease of use, accuracy, consistency, reliability, and reproducibility in evaluating spine and pelvis conditions when transitioning from the *AMA Guides to the Evaluation of Permanent Impairment (AMA Guides)* Sixth Edition 2008 to the newly updated Sixth Edition 2024. **Methods**: Two rounds of impairment ratings were performed by a team consisting of three physician experts and four premedical students, focusing on a comparison between the 2008 and 2024 editions of the *AMA Guides*. The analysis included both the impairment values generated and the time taken to complete assessments with each version. **Results**: For the expert group, the mean duration required to complete an impairment rating was 5.0 min with the *AMA Guides* 2024, compared to 15.4 min using the *AMA Guides* 2008, with both editions achieving 100% accuracy and reliability. The premedical students demonstrated similar improvements, averaging 8.4 min per rating with the 2024 edition versus 26.4 min with the 2008 edition. The *AMA Guides* 2024 yielded enhanced accuracy, consistency, reliability, and reproducibility. **Conclusions**: The *AMA Guides* Sixth Edition 2024 represents a significant advancement in impairment evaluation, particularly for spine and pelvis assessments. This updated edition introduces a more streamlined and time-efficient process while preserving the accuracy, consistency, and reproducibility essential to high-quality impairment ratings. By enhancing clarity and standardization, it sets a new standard in occupational health, offering a reliable framework that supports both clinical assessment and administrative oversight.

## 1. Introduction

The *AMA Guides to the Evaluation of Permanent Impairment* (*AMA Guides*) serve as the most commonly utilized framework in both the United States and globally for assessing functional loss—commonly referred to as impairment—resulting from injury or disease. [1,2]. Independent medical examinations (IMEs) play a critical role in numerous legal systems for establishing the presence of a compensable injury [3]. The independent medical evaluator holds a key responsibility in verifying the existence of a permanent medical condition and determining the level of impairment based on objective and reproducible evidence drawn from the clinical history, physical examination, and relevant clinical studies [1,2]. These impairment values serve as benchmarks within administrative and legal frameworks to guide appropriate monetary compensation.

The *AMA Guides* Sixth Edition 2008 faced criticism for its steep learning curve, extensive training requirements, inconsistent definitions, and concerns over reliability, reproducibility, and content validity [4,5,6,7,8]. However, quality IME reports remain essential, benefiting all stakeholders through thorough evaluations by physicians with both clinical expertise and medicolegal proficiency [9].

To address noted concerns, the AMA launched a comprehensive review of the Guides and, in June 2019, created the *AMA Guides* Editorial Panel (Guides Panel) to update the content in line with current developments in diagnosis, treatment, outcome measurement, functional assessment, and impairment evaluation. In response to stakeholder input, the Guides Panel established a Musculoskeletal (MSK) subcommittee in August 2022 to reexamine material related to the upper and lower limbs, as well as the spine and pelvis. Their collaborative efforts resulted in major updates, including the use of the RAND/UCLA modified Delphi Method, public comment periods, and a new five-step impairment evaluation process [10].

The *AMA Guides* Sixth Edition 2024 introduces enhanced diagnosis-based impairment (DBI) tables that integrate specific individual elements (SIEs) from the clinical history, physical examination, and relevant studies, ensuring alignment with modern medical practices. The 2024 edition improves ease of use, accuracy, consistency, and reliability, addressing prior limitations and setting a new standard for spine and pelvis assessments.

The purpose of this study was to evaluate the methodological improvements introduced in the *AMA Guides to the Evaluation of Permanent Impairment* Sixth Edition 2024, specifically within the musculoskeletal chapters. The study compared the updated 2024 edition with the 2008 edition, focusing on several key outcomes: ease of use, accuracy, interrater and intrarater reliability, consistency, and reproducibility.

To guide this evaluation, the study posed four central research questions: (1) Does the 2024 edition improve ease of use compared to the 2008 edition?; (2) Are impairment ratings using the 2024 edition more accurate and consistent than those derived from the 2008 edition?; (3) Does the 2024 edition enhance interrater and intrarater reliability?; and (4) Can the updated methodology reduce evaluation time without compromising accuracy or reproducibility?

These questions informed the development of four corresponding hypotheses. H1 proposed that the 2024 edition would significantly reduce the time required to complete impairment ratings. H2 proposed that the updated edition would result in more accurate ratings, including among less experienced evaluators such as premedical students. H3 hypothesized that the revised method would yield greater interrater and intrarater reliability due to its simplified, standardized structure. Finally, H4 suggested that the enhanced diagnosis-based impairment (DBI) tables and the structured five-step sequential method would improve the overall consistency and reproducibility of impairment evaluations.

## 2. Materials and Methods

The study was designed and documented in accordance with the Guidelines for Reporting Reliability and Agreement Studies (GRRAS) [11].

The *AMA Guides* update process included the appointment of author–editors for the musculoskeletal (MSK) chapters and the formation of a spine subcommittee [12]. In collaboration with the North American Spine Society (NASS), the spine subcommittee revised content from the 2008 edition to align with current diagnoses, treatments, and outcomes that were previously unavailable or uncommon. To ensure scientific rigor, the subcommittee applied the RAND/UCLA Appropriateness Method (RAM) with a modified Delphi process to critically evaluate research on impairment evaluations [13].

The Delphi technique is widely recognized as an effective method for reaching consensus on complex or debated topics [14]. Its strength lies in key features such as respondent anonymity, iterative rounds of structured questionnaires, minimized influence from dominant individuals or group dynamics, and the provision of controlled feedback between rounds [14,15,16]. The process typically begins with an open-ended or structured questionnaire designed to uncover essential themes related to the topic [14]. In the subsequent round, participants are asked to rate or prioritize the identified elements based on perceived importance. This cycle is repeated in successive rounds until a sufficient level of agreement—or consensus—is achieved among the panel members [14].

**Figure 1:**
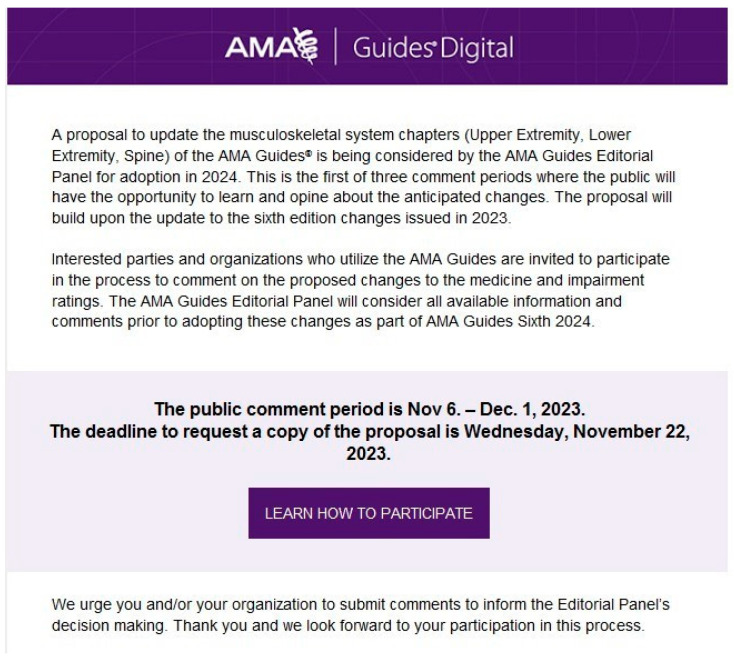
Example of the AMA Guides Digital Public Comment. Reprinted from Melhorn J.M., Gelinas B., Martin D.W., et al. *Advancements in AMA Guides Musculoskeletal Impairment Evaluations: Improved Reliability and Ease of Use. J Occup Environ Med.* Published online 10 May 2024. Doi:10.1097/JOM.0000000000003145. © 2024 Wolters Kluwer Health, Inc. Reprinted with permission [20].

**Figure 2:**
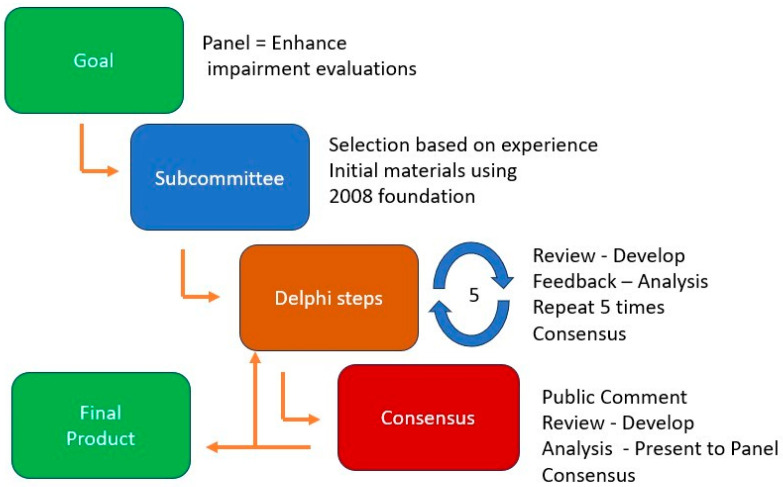
The RAND/UCLA Delphi Method. Reprinted from Melhorn J.M., Gelinas B., Martin D.W., et al. *Advancements in AMA Guides Musculoskeletal Impairment Evaluations: Improved Reliability and Ease of Use. J Occup Environ Med.* Published online 10 May 2024. DOI:10.1097/JOM.0000000000003145. © 2024 Wolters Kluwer Health, Inc. Reprinted with permission [20].

Once the foundational content was established, the subcommittee sought input from a broad group of stakeholders, including administrative law judges, attorneys, chiropractors, disability evaluators, neurologists, surgeons, pain specialists, psychiatrists, psychologists, and state workers’ compensation officials. This diverse feedback informed chapter updates, which were further refined through multiple public comment periods (Figure 1). The final version of the chapter, incorporating insights from reviewers and contributors, underwent several Delphi method reviews before receiving approval from the Guides Panel (Figure 2) [17,18,19].

The MSK subcommittee’s first objective was to revise and streamline the impairment evaluation process by defining the essential steps for determining a rating. Through a consensus-driven approach and the application of the RAND/UCLA Appropriateness Method (RAM), these steps were condensed into five, simplifying the overall process. This refinement integrates the concepts of class, grade, and impairment value into a single diagnostic row, incorporating specific individual elements (SIEs) from the clinical history, physical examination, and relevant clinical studies (see Table 1). Feedback from multiple stakeholders further shaped this streamlined approach, enhancing the efficiency and clarity of the evaluation method.

The second objective aimed to improve the diagnosis-based impairment (DBI) tables in the three musculoskeletal (MSK) chapters by incorporating specific individual elements (SIEs) derived from the clinical history, physical examination, and relevant clinical studies. These SIEs offer verifiable, objective criteria for diagnosis, as well as valuable insights into outcomes and functional losses. Examples of SIEs from the physical examination include sensory deficits, muscle strength grading, reflex changes, limb atrophy, and nerve tension signs. Relevant clinical studies include imaging modalities such as radiographs, MRI, CT, and ultrasound, as well as electrodiagnostic tests and laboratory evaluations. To implement these enhancements, the spine subcommittee utilized a five-round modified Delphi method (Figure 2), drawing upon diagnostic categories and impairment values from the 2008 edition. This structured, consensus-based process allowed for a quantitative evaluation of key diagnoses and SIEs necessary for accurate impairment assessments within the revised DBI tables.

The enhanced DBI tables for the spine and pelvis are structured to integrate smoothly with the evaluator’s approach to impairment assessment. The process starts with the evaluator identifying specific individual elements (SIEs) from the clinical history, physical examination, and relevant clinical studies, ensuring an accurate diagnosis. The identified diagnosis then directs the evaluator to the appropriate DBI table for additional assessment.

For instance, an individual reports residual symptoms consistent with right L4 radiculopathy. His physical examination reveals a sensory deficit, including decreased sharp vs. dull perception (reduced protective sensibility) in the dermatomal distribution consistent with the L4 dermatome, which includes the anterolateral thigh, crossing the knee, and extending to the medial dorsum of the foot and the big toe. Magnetic resonance imaging confirms involvement of the right L4 nerve root. These SIEs align with diagnostic row 17-21-06, class 1C, corresponding to a 5% whole person impairment (WPI), as outlined in Table 2.

The third objective focused on evaluating the impact of the four study hypotheses by comparing the *AMA Guides* Sixth Edition 2024 compared to the 2008 edition, using illustrative case vignettes to evaluate impairment ratings across various diagnoses. These vignettes, aligned with the 2008 DBI table criteria, simplify scenarios with limited clinical context based on the clinical history, physical examination, and relevant studies. While this simplification streamlines the process, it underestimates the time and effort required for comprehensive data gathering and interpretation during real-world evaluations. As noted in the *AMA Guides* 2008 statement #8), the evaluating physician must use knowledge, skill, and ability generally accepted by the medical scientific community when evaluating an individual to arrive at the correct impairment rating according to the Guides [11].

This approach aims to bridge knowledge gaps and enhance nonmedical stakeholders’ understanding of the methodology and rationale behind impairment ratings using the streamlined five-step method in the updated edition. To assess the differences between the two editions, the spine subcommittee randomly selected five case examples from the 2008 edition for comparison. The evaluation tested ease of use (time to complete the rating), accuracy (agreement with published values), consistency (reproducibility of results), reliability (consistency over time), and reproducibility (both intrarater and interrater). Three physician experts from the Guides Panel and four premedical students participated in the comparison.

The four individuals included in this study were premedical students, not enrolled in medical school at the time of participation. They were selected to represent individuals with minimal prior exposure to impairment evaluation, allowing us to assess the ease of use and learning curve of the two editions of the *AMA Guides* from a novice perspective.

The participants were recruited through voluntary outreach from a university premedical advising program and were not part of a formal or themed class on occupational injury or impairment evaluation. Their academic standing was fourth-year undergraduate students, with a shared interest in medicine but no prior experience using the *AMA Guides*. This cohort was intentionally selected to help demonstrate usability differences between the editions among users with little to no background in formal clinical assessment.

Three physician experts and four premedical students were given the 2008 edition of the *AMA Guides* Sixth Edition—excluding case examples—along with step-by-step instructions and sample data to complete impairment assessments for five test cases. They then repeated the process using the steps and enhanced DBI tables from the *AMA Guides* Sixth Edition 2024. Given the premedical students’ limited experience with impairment ratings, they received additional guidance and could ask questions before performing their evaluations.

To mitigate potential learning effects from the fixed order of exposure (2008 edition followed by 2024 edition), several methodological safeguards were implemented. Evaluators repeated the impairment ratings eight weeks later, using the same cases presented in randomized order for each participant. This interval and randomization helped minimize recall and sequence bias, preserving the integrity of intrarater and interrater reliability. Additionally, this second round increased the number of data points, further strengthening the study’s statistical analysis and overall findings.

Normality testing revealed that the data did not meet the required assumptions, with skewness and kurtosis values exceeding 1.0. As a result, comparisons between the 2008 and 2024 editions of the *AMA Guides* Sixth Edition were performed using Wilcoxon rank-sum tests. Interrater reliability was evaluated using kappa statistics. All statistical analyses were conducted at a significance threshold of 0.05 using SAS software, version 9.4 (SAS Institute, Cary, NC, USA).

## 3. Results

### 3.1. Time to Complete Rating (Ease of Use)

The three physician experts completed ratings using the *AMA Guides* 2008 in an average of 15.4 min. With the *AMA Guides* 2024, this time was reduced to just 5.0 min—representing a 67.5% time saving—while maintaining 100% accuracy or reliability in both rounds (Table 3). Cohen’s Kappa, calculated with SAS, was 1.0 for both interrater and intrarater reliability [21,22].

For the four premedical students, the average completion time was 26.4 min using the 2008 edition, compared to 8.4 min with the 2024 edition, reflecting a 68.2% time reduction (Table 4). The longer time with the 2008 edition resulted from navigating non-key factor tables to apply functional history, physical examination, and clinical study adjustments within the net adjustment formula. The original 2008 case examples were intentionally designed to align with the 2008 impairment assessment criteria, thereby minimizing the need for premedical students to engage in deeper clinical reasoning to identify the “preferred” grade modifiers—mild, moderate, or severe—as outlined in the grade modifier tables. It is important to note that these simplified examples do not reflect the complexity typically encountered by professional evaluators when conducting actual impairment ratings.

The time required for both physicians and students remained consistent across the second round, indicating minimal learning benefit from prior exposure. Statistically significant differences in the completion times were found between the 2008 and 2024 editions (*p* < 0.0001) for both groups, with results favoring the 2024 edition. These differences persisted when analyzed separately for experts and students (*p* < 0.0001 for each).

### 3.2. Accuracy

Accuracy was defined as correctly matching the published impairment values from the 2008 edition and the expert panel members’ (EPMs’) determinations for the 2024 edition. One exception was made for 2008 example case 17-3. Although the Sixth Edition lists this example as an 11% whole person impairment (WPI), all the expert panel members independently concluded the correct rating should be a 10% WPI when applying the 2008 methodology. This discrepancy underscores the inherent complexity of the 2008 rating process. While the default impairment for grade C is an 11% WPI, proper application of the grade modifiers results in a net adjustment of −1, yielding a final rating of 10% WPI, consistent with grade B. For this reason, a 10% WPI was used as the reference standard for student accuracy.

Table 5 presents the accuracy results for both round 1 and round 2, which took place eight weeks later. A value of 1 indicates concordance with the reference impairment value, while a value of 0 denotes a discrepancy.

In round 1, for the *AMA Guides* 2008, one of the four premedical students (PMS1) rated three of the five examples correctly, whereas three of the four premedical students rated one of the five correctly. For the *AMA Guides* 2024, all four students rated all five examples correctly.

In round 2, for the *AMA Guides* 2008, one of the four premedical students (PMS1) rated two of the five examples correctly, one of the four premedical students rated one of the five examples correctly, and two of the four premedical students rated 0 of the five correctly. For the *AMA Guides* 2024, all four premedical students rated all five examples correctly. The variability in impairment ratings for the *AMA Guides* 2008 stemmed from the reliance on modifier tables that categorized values as mild, moderate, or severe, rather than on specific individual elements (SIEs). In contrast, the accuracy of the *AMA Guides* 2024 was driven by the use of anatomical SIEs outlined in the physical examination criteria and relevant clinical studies, which aligned with the diagnostic row and corresponding impairment value in the DBI table.

Across both rounds, the *AMA Guides* 2024 produced significantly more accurate ratings than the 2008 edition (*p* < 0.0001) (Table 5).

### 3.3. Consistency, Reliability, and Reproducibility

Consistency refers to the repeatability of results under the same conditions, forming part of reliability, which reflects the overall dependability of a measurement process. Reliability includes consistency, the absence of random errors, and reproducibility across different evaluators or conditions, often assessed through test–retest, interrater, and intrarater reliability. Reproducibility ensures that the same results can be achieved across various observers, instruments, or protocols, confirming that a process can be reliably duplicated.

Using both the 2008 and 2024 methodologies, the physicians reported the correct impairment values for all five examples across both rounds. However, the data from the premedical students highlight the challenges less experienced evaluators face in achieving consistent, reliable, and reproducible ratings with the *AMA Guides* 2008 (Table 6).

The following results can be noted:

- One student correctly matched two examples and incorrectly matched two in both rounds but reported one correct in round 1 that was incorrect in round 2;
- Another student matched one example correctly and four incorrectly in both rounds;
- Two students each had one correct match in round 1 but none in round 2.

In contrast, all four students reported the correct impairment values for all five examples using the 2024 method in both rounds, demonstrating greater consistency, reliability, and reproducibility. The Kappa statistic for the 2008 edition was 0.583, indicating moderate to good reliability, compared to a perfect 1.00 for the 2024 edition. This difference is statistically significant (*p* < 0.01), confirming that the agreement with the *AMA Guides* 2024 is significantly better than with the 2008 edition.

## 4. Discussion

This study represents a novel and methodologically rigorous evaluation of the 2024 updates to the AMA Guides to the Evaluation of Permanent Impairment Sixth Edition, specifically focused on the musculoskeletal (MSK) chapters. While previous editions of the AMA Guides have faced criticism regarding complexity, low reproducibility, and steep learning curves, this is the first published research to systematically compare the 2008 and 2024 editions using quantifiable outcome measures such as ease of use, accuracy, interrater and intrarater reliability, and reproducibility.

Moreover, the research employs an innovative dual-participant model involving both expert evaluators and novice users (premedical students)—a distinctive approach that broadens the scope of the evaluation to include real-world stakeholders who may interact with impairment ratings indirectly. This inclusion of novice evaluators adds a valuable dimension to assessing the user-friendliness and accessibility of the revised Guides.

Additionally, the study integrates a modified Delphi consensus process and applies the RAND/UCLA Appropriateness Method, providing a structured, evidence-informed pathway for guideline development rarely seen in this domain.

The significance of this research lies in its potential to transform impairment rating practices in both clinical and legal contexts. The AMA Guides are widely used in the U.S. and internationally for determining permanent impairment and guiding compensation decisions. Improving the clarity, efficiency, and reliability of these evaluations has direct implications for fairness in compensation, administrative efficiency, and medicolegal accuracy.

The updated method and enhanced DBI tables in the *AMA Guides* Sixth Edition 2024 represent significant advancements in the ease of use, accuracy, consistency, and reliability of spine and pelvis impairment ratings. Similar consistency improvements have been reported for upper limb conditions, reinforcing the universal applicability of these updates across various body regions [20]. These enhancements reduce the need for extensive evaluator training and increase the reproducibility of ratings, aligning with previous findings [20].

This study confirms that the updated method and DBI tables significantly improve the standardized rating process for spine and pelvis evaluations by enhancing ease of use, accuracy, consistency, reliability, and reproducibility (both interrater and intrarater). The *AMA Guides* 2024 are more user-friendly, offering particular advantages for novice evaluators, with minimal learning required.

The spine subcommittee used a modified Delphi process to develop the updated method and enhanced DBI tables while maintaining the AMA’s criteria for fair and equitable impairment ratings based on objectively verifiable anatomical or physiological findings. Requiring evaluators to follow all five steps (Table 1) improved the outcome measures, including time to completion, accuracy, consistency, reliability, and reproducibility.

The physician experts evaluated five example cases using clinical data from the *AMA Guides* 2008, achieving 100% accuracy, consistency, reliability, and reproducibility with both the 2008 and 2024 methods. However, the 2024 method reduced the average completion time by 10.4 min (15.4 vs. 5.0 min), yielding a 67.5% time saving, even for experienced evaluators familiar with the older edition.

Concerns with the 2008 edition included complexity, lengthy completion times, lower accuracy (due to navigating multiple tables), and reduced reproducibility. To assess the impact of these challenges, the premedical students also completed the ratings. As expected, the 2024 method improved ease of use, reducing completion time by 18.0 min (26.4 vs. 8.4 min) for the students—a 68.2% time saving—while also enhancing accuracy, consistency, and reliability, with no noticeable learning curve.

These findings suggest that the *AMA Guides* 2024 not only streamline the impairment rating process but also improve the quality and reliability of the evaluations, making the Guides an optimal tool for both experienced and novice evaluators. Additionally, these improvements are likely to enhance the interpretation of reports by stakeholders such as administrative law judges, further increasing the practical value of the updated edition.

The five-step methodology introduced in the AMA Guides 2024 provides a systematic structure for producing accurate and appropriate impairment ratings. Its clearly defined, sequential documentation supports transparency, enhances understanding, and enables effective quality assurance and review. Compared to the 2008 edition, the 2024 method reduces the need for extensive evaluator training by integrating core healthcare elements such as the clinical history, physical examination, and relevant clinical studies. Its improved consistency and ease of use are expected to yield cost savings for the workers’ compensation system. However, the full realization of these benefits will depend on acceptance and implementation within the appropriate legislative or jurisdictional frameworks.

The primary limitation of this study is its reliance on clinical vignettes. While robust study designs that evaluate impairment ratings for heterogeneous and complex patient presentations would be ideal, they are challenging to implement. While we acknowledge that these ratings are based on consensus and expert opinion and that a larger and more diverse pool of evaluators could further strengthen generalizability, the purpose of this study was to conduct a targeted, controlled comparison of the methodological changes introduced in the *AMA Guides* Sixth Edition 2024 relative to the 2008 edition.

Given that limitation, future research could entail more comprehensive studies involving heterogeneous and complex patient presentations, which would provide greater insight, though their implementation poses significant challenges. It is important to recognize that the impairment ratings in this study are derived from consensus and expert opinion.

Moreover, real-world patients often present with diverse conditions, varying degrees of impairment for similar diagnoses, and complex medical histories, along with numerous influencing factors [23,24,25]. These complexities are further compounded by the inherent variability in individual patient responses, making standardized assessments more challenging.

It is important to clarify that the AMA Guides are designed to establish impairment ratings, not to determine compensation or disability outcomes. Final decisions regarding compensation or disability are made by the relevant adjudicating authority. While the Guides focus solely on evaluating impairment, disability determinations incorporate broader considerations such as age, daily functional abilities, educational background, occupational demands, regional context, workplace accommodations, available social support, and overall community impact—factors that go beyond the scope of impairment assessment.

## 5. Conclusions

This research offers clear, real-world application value by demonstrating that the *AMA Guides* Sixth Edition 2024 provides a significantly more efficient, accurate, and reliable method for evaluating musculoskeletal impairments compared to the 2008 edition. These improvements are especially critical for the wide range of stakeholders who rely on impairment ratings to make informed decisions related to medical care, disability determination, and compensation.

The 2024 edition introduces substantial updates to the spine and pelvis chapter, with changes grounded in contemporary medical science and practical evaluator feedback. Developed using a modified Delphi method, the revised framework incorporates quality measures and structured algorithms, which address longstanding issues of ambiguity, inconsistency, and low reproducibility seen in the 2008 edition.

Importantly, the 2024 edition reflects a shift toward evidence-based, standardized assessments, with a clear focus on objectively verifiable clinical elements—including patient history, physical examination, and relevant clinical studies. This alignment with real-world clinical workflows not only enhances usability but also streamlines evaluator training, making the methodology accessible to both experienced physicians and novice users, as demonstrated by the strong performance of premedical students in this study.

Furthermore, the improved consistency and clarity of the rating process has the potential to reduce administrative burden and costs in systems such as workers’ compensation, provided that the updated *Guides* are embraced by relevant jurisdictions and legislative bodies. These benefits directly respond to concerns about the time-intensive, error-prone nature of the 2008 edition, which the current study set out to investigate.

In summary, this study validates the application and utility of the *AMA Guides* Sixth Edition 2024 as a major advancement in the field of impairment evaluation. By empirically assessing its performance against the 2008 edition—specifically in terms of ease of use, accuracy, interrater and intrarater reliability, and reproducibility—the study confirms that the updated methodology not only improves the quality of impairment ratings but also aligns with the evolving demands of clinical practice, legal systems, and health policy. These findings fulfill the original purpose of the study and address the key research questions, demonstrating the practical and policy-relevant impact of the 2024 revision.

## Figures and Tables

**Table 1:** The 2024 Five-Step Process for Spine and Pelvis Impairment Rating.

2024 AMA Guides Musculoskeletal Impairment Rating Steps	
Step 1.	Confirm a Clinically Relevant Diagnosis (DX)
Step 2.	Confirm Maximum Medical Improvement (MMI)
Step 3.	Identify the Relevant Diagnosis-Based Impairment (DBI) Table
Step 4.	Determine the Diagnostic Row, Class, Grade, and Impairment Value
Step 5.	Guidelines for Report Documentation

**Table 2:** Example of Diagnostic Row in Spine DBI Table.

Impairment Criteria	Details
DBI Table	17-21-06 Lumbar Radiculopathy Involving the L4 Nerve Root
Impairment Class	Class 1C
Whole Person Impairment (WPI)	5%
Clinical History (CH)	Residual symptoms with a mechanism of injury consistent with the diagnosis
Physical Examination (PE)	Sensory deficit—loss of sharp vs. dull perception (decreased protective sensibility) in the L4 dermatomal distribution (anterolateral thigh, crossing the knee to medial dorsum of foot, big toe)
Clinical Studies (CSs) (*one of the following*)	- MRI or CT imaging demonstrating an L4 nerve root injury or lesion
	- Electrodiagnostic findings confirming L4 nerve root pathology consistent with Guides CS definitions)
	- Surgeon’s clear objective verification of an intraoperative lesion involving the L4 nerve root

**Table 3:** Average Time to Complete Impairment Ratings, AMA Guides 2008 vs. Guides 2024, by Expert Panel Members.

Round 1	EPM1		EPM2		EPM3	
Examples	2008	2014	2008	2014	2008	2014
1	18	6	18	6	19	6
2	21	5	20	5	21	5
3	8	4	7	3	7	4
4	22	6	21	6	20	6
5	10	5	9	3	8	4
Average time, min *	15.8	5.2	15	4.6	15	5
**Round 2**	**EPM1**		**EPM2**		**EPM3**	
**Example**	**2008**	**2014**	**2008**	**2014**	**2008**	**2014**
1	18	6	19	6	20	5
2	21	5	22	5	21	6
3	7	4	6	3	6	4
4	23	6	22	6	21	6
5	10	5	9	4	8	5
Average time, min ^#^	15.8	5.2	15.6	4.8	15.2	5.2

* Round 1 combined average time (min) for all EPMs: AMA Guides 2008, 15.2; AMA Guides 2024, 4.9; ^#^ Round 2 combined average time (min) for all EPMs: AMA Guides 2008, 15.5; AMA Guides 2024, 5.0; EPM: indicates expert panel member.

**Table 4:** Average Time to Complete Impairment Ratings, AMA Guides 2008 vs. Guides 2024, for the premedical students.

Round 1	PMS1		PMS2		PMS3		PMS4	
Example	2008	2024	2008	2024	2008	2024	2008	2024
1	28	10	29	9	31	9	30	9
2	29	7	34	9	30	10	30	10
3	10	5	21	6	20	7	19	6
4	35	8	30	10	31	10	29	10
5	15	8	26	8	24	7	25	9
Average time, min *	23.4	7.6	28	8.4	27.2	8.6	26.6	8.8
**Round 2**	**PMS1**		**PMS2**		**PMS3**		**PMS4**	
**Example**	**2008**	**2024**	**2008**	**2024**	**2008**	**2024**	**2008**	**2024**
1	19	8	30	9	32	10	31	10
2	29	8	33	8	31	9	30	9
3	20	5	20	6	20	6	19	6
4	29	9	30	9	32	11	30	10
5	22	8	24	9	25	8	24	10
Average time, min ^#^	23.8	7.6	27.4	8.2	28	8.8	26.8	9.0

* Round 1 combined average time (min) for all PMSs: *AMA Guides* 2008, 26.3; *AMA Guides* 2024, 8.4; ^#^ Round 2 combined average time (min) for all PMSs: *AMA Guides* 2008, 26.5; *AMA Guides* 2024, 8.4; PMS: indicates premedical student.

**Table 5:** Accuracy of *AMA Guides* 2008 vs. *AMA Guides* 2024 for the Premedical Students.

Round 1	2008	2008	2008	2008	2024	2024	2024	2024
Example	PMS1	PMS2	PMS3	PMS4	PMS1	PMS2	PMS3	PMS4
1	1	0	0	0	1	1	1	1
2	0	0	0	0	1	1	1	1
3	1	1	1	1	1	1	1	1
4	0	0	0	0	1	1	1	1
5	1	0	0	0	1	1	1	1
Number correct	3	1	1	1	5	5	5	5
**Round 2**	**2008**	**2008**	**2008**	**2008**	**2024**	**2024**	**2024**	**2024**
**Example**	**PMS1**	**PMS2**	**PMS3**	**PMS4**	**PMS1**	**PMS2**	**PMS3**	**PMS4**
1	0	0	0	0	1	1	1	1
2	0	0	0	0	1	1	1	1
3	1	0	0	0	1	1	1	1
4	0	0	0	0	1	1	1	1
5	1	1	0	0	1	1	1	1
Number correct	2	1	0	0	5	5	5	5

PMS: indicates premedical student.

**Table 6:** Consistency, Reliability, and Reproducibility of the AMA Guides 2008 vs. the AMA Guides 2024 *.

	Round 1	Round 2	Round 1	Round 2	Round 1	Round 2	Round 1	Round 2
	2008	2008	2008	2008	2008	2008	2008	2008
Example	PMS1	PMS1	PMS2	PMS2	PMS3	PMS3	PMS4	PMS4
1	1	1	0	0	0	0	0	0
2	0	0	0	0	0	0	0	0
3	1	1	0	0	1	0	1	0
4	0	0	0	0	0	0	0	0
5	1	0	1	1	0	0	0	0
Overall number correct	3	2	1	1	1	0	1	0

* Data from 2024 were 5/5 correct and 5/5 correctly matched for all assessments; PMS: indicates premedical student.

## Data Availability

The original contributions presented in this study are included in the article. Further inquiries can be directed to the corresponding author.

## Abbreviations

The following abbreviations are used in this manuscript:

AMA	American Medical Association
IME	Independent medical examination
MSK	Musculoskeletal
DBI	Diagnosis-based impairment
SIE	Specific individual element
WPI	Whole person impairment
EPM	Expert panel member
PMS	Premedical student
